# Vitamin D in the Prevention and Treatment of Osteoarthritis: From Clinical Interventions to Cellular Evidence

**DOI:** 10.3390/nu11020243

**Published:** 2019-01-22

**Authors:** Clara Yongjoo Park

**Affiliations:** Department of Food and Nutrition, Human Ecology Research Institute, Chonnam National University, Gwangju 61186, Korea; parkcy@jnu.ac.kr; Tel.: +82-62-530-1354

**Keywords:** vitamin D, osteoarthritis, initiation, progression, review

## Abstract

Older adults are recommended vitamin D to prevent fractures. Though this population is also at risk of osteoarthritis (OA), the effect of vitamin D on OA is unclear and may differ by disease state. The relationship between vitamin D and OA during OA initiation and progression were considered in this narrative review of in vivo and in vitro studies. Regarding OA initiation in humans, the small number of published observational studies suggest a lack of association between induction of OA and vitamin D status. Most randomized controlled trials were performed in White OA patients with relatively high vitamin D status (>50 nmol/L). These studies found no benefit of vitamin D supplementation on OA progression. However, subset analyses and one randomized controlled pilot trial indicated that vitamin D supplementation may alleviate joint pain in OA patients with low vitamin D status (<50 nmol/L). As the etiology of OA is recently being more fully uncovered, better animal and cell models are needed. According to currently available clinical results, evidence is lacking to set a vitamin D level to prevent OA, and increasing vitamin D status above 50 nmol/L does not seem to benefit OA patients.

## 1. Introduction

Vitamin D is known to increase bone mass or prevent bone loss. Thus, older adults are recommended to consume adequate vitamin D to prevent osteoporosis and fracture [1]. Vitamin D insufficiency is a global nutrition challenge, especially for institutionalized older persons [2]. Public awareness of the necessity of vitamin D has increased in recent decades. For instance, the use of high-dose vitamin D supplements (≥1000 International Units (IU)/d) is increasing in the United States (US) with over 30% of adults aged ≥60 years consuming ≥1000 IU/d through supplementation [3]. The new US Nutritional Facts Label provides the actual amount and percent of the daily recommended intake of vitamin D contained in food products in order to increase awareness for vitamin D intake [4]. As more attention is focused on vitamin D intake, the effect of higher vitamin D status, in addition to low vitamin D status, on other health outcomes needs to be established to prevent possible negative health consequences from high vitamin D status and to establish the level of adequacy for optimal health.

The aging population is at risk for not only osteoporosis, but also osteoarthritis (OA). In the US, 30 million adults are affected by OA [5]. Symptomatic knee OA occurs in 10% of men and 13% of women aged ≥60 years [6]. As the proportion of older adults increases in developed nations, OA is likely to affect a greater percentage of the population in future decades. Pain and decreases in motor ability due to OA can result in higher risk of obesity, diabetes, falls, and fracture [7]. Currently, joint replacement surgery, usually performed after years of enduring pain, is the only widely available treatment for OA [8]. Although this pandemic has been ongoing for centuries, the etiology of OA has started to unfold relatively recently. Though previously OA was known to be a ‘wear-and-tear’ disease, the current understanding is that many factors, in addition to old age and mechanical stress, may induce OA [8]. In addition, evidence points to potential OA pathways that vitamin D may interact with, though the relationship may depend on stage of OA (initiation or progression). For instance, greater bone mass, which is associated with adequate vitamin D status, may be positively associated with the initiation and progression of OA [9]. Few studies have determined the effect of vitamin D on OA initiation. The causal effect of vitamin D on OA progression tends to point to a protective effect in patients with low vitamin D status. Therefore, understanding the effect of vitamin D on OA is critical, especially in older adults who are recommended to increase vitamin D intake to prevent osteoporosis and fractures. This narrative review focuses on the potential relationship between vitamin D and OA by considering the available clinical data in addition to cellular and animal data.

## 2. Current Status of Knowledge

### 2.1. Vitamin D

The 2011 Institute of Medicine (IOM) recommendations for vitamin D intake are based on evidence for bone health (calcium absorption, bone mineral density, and vitamin D deficiency rickets/osteomalacia) [1]. Vitamin D can be obtained through foods such as fatty fish, mushrooms, and vitamin D-fortified products, and through cutaneous synthesis in response to ultraviolet-B exposure. Vitamin D is bound to the vitamin D binding protein (DBP) and metabolized in the liver to 25-hydroxyvitamin D (25(OH)D), the marker for vitamin D status due to its relatively long half-life (3–4 weeks) [10]. In the endocrine system, serum 25(OH)D is converted to the active vitamin D metabolite, 1,25-dihydroxyvitamin D (1,25(OH)_2_D), by renal 1α-hydroxylase (1αOHase). Activation of renal 1αOHase is induced by parathyroid hormone (PTH) when low levels of serum calcium (Ca) are detected by the parathyroid gland. Circulating 1,25(OH)_2_D binds to the vitamin D receptor (VDR), resulting in heterodimerization of the VDR with the retinoic X receptor (RXR) and subsequent translocation to the nucleus [11]. The increase in serum 1,25(OH)_2_D, in turn, negatively regulates PTH. In addition to dietary intake and sun exposure, vitamin D status itself is influenced by genetics, especially single nucleotide polymorphisms of genes encoding DBP, 25-hydroxylase, and 1αOHase [12,13]. The classic role of vitamin D is known to increase Ca absorption through the endocrine pathway. In the small intestine, vitamin D induces the transcription of calcium transport genes *TRPV6*, *Calbindin D_9k_*, and *PMCA1b*, resulting in increased active Ca absorption [11]. However, as more precise methods for Ca absorption measurement have been developed over recent decades, the effect of vitamin D on Ca absorption in adults seems minimal, especially in adults with 25(OH)D levels ≥ 20 nmol/L [14,15,16,17] and does not increase calcium absorption in adolescents [18,19]. However, vitamin D supplementation in those with 25(OH)D <50 nmol/L appears to increase bone mass or prevent bone loss in adults [20,21] and positively affect bone mineral augmentation in adolescents [22,23]. Therefore, it is proposed that vitamin D may benefit bone through an autocrine and/or paracrine pathway, especially since circulating 1,25(OH)_2_D, the active vitamin D metabolite, is not associated with 25(OH)D status. A more thorough review on vitamin D metabolism and bone can be found elsewhere [24]. Though the mechanism by which vitamin D affects bone is unclear, serum 25(OH)D ≥50 nmol/L is required for optimal bone health.

At the time of the literature review by the IOM committee, OA was not included among the potential indicators of adequacy, which included cancer/neoplasms, cardiovascular diseases and hypertension, diabetes and metabolic syndrome, falls and physical performance, and immune responses [1]. However, during the past decade, several studies have been published on the relationship between vitamin D and OA, increasing our knowledge on this subject.

### 2.2. Osteoarthritis

Characterization of OA includes joint pain, joint space narrowing (JSN), osteophyte formation, and cartilage damage [25]. However, standard definitions are still lacking [25]. Until recently, OA was thought to be a ‘wear-and-tear’ disease, unable to prevent or treat besides limiting the use of joints and temporarily relieving pain. Patients with severe OA may be eligible for joint replacement surgery, which can provide pain relief for approximately 20 years. This results in a narrow age range of eligibility for surgery. Stem cell therapy is currently in its developmental stage, which may be beneficial for patients who fail to respond to medical treatment or are unable to undergo surgery, although opinions on this method are conflicting [26,27]. Research in recent decades concerning the etiology of OA may expedite the development of new methods to prevent and treat OA.

Articular cartilage and its underlying subchondral bone are highly affected by OA. These regions consist of osteoblasts, osteoclasts, osteocytes, and articular chondrocytes. Articular chondrocytes differ from growth plate chondrocytes in that they are fully differentiated cells in resting state that rarely proliferate and hypertrophy does not occur [28]. Further differentiation or hypertrophy of articular chondrocytes is a phenotype of OA. Osteoarthritic joints have low levels of aggrecan, proteoglycan, type-II collagen, and runt-related transcription factor 1(RUNX1). In contrast, type-X collagen, matrix metalloproteinase (MMP) 13, and a disintegrin and metalloproteinase with thrombospondin motifs (ADAMTS) 5, are elevated and used as molecular markers of OA. Other characteristics of OA include disorientation, calcification, and vascular penetration of the articular cartilage, destruction of subchondral bone, JSN, and formation of osteophytes. Radiological OA is usually assessed in clinical settings based on the existence of JSN and osteophytes. Patients may also experience pain, swelling, and loss of function in the joints. OA can be detected by various methods, ranging from protein expression at the molecular level to radiography and imaging in clinical settings.

Among the molecular pathways involved with OA, which have been extensively reviewed previously [29], the transforming growth factor-beta (TGFβ)/SMAD pathway may be involved with vitamin D [30,31,32]. The role of TGFβ may depend on the condition of the joint [30,31,32,33]. In healthy joints, TGFβ protects the joint. Normally, TGFβ binds to its receptor and phosphorylates SMAD3, which represses MMP13 expression [33]. In healthy mouse joints, blockage of TGFβ results in distorted repair responses in the joint [34]. Lack of TGFβ3 is associated with cartilage damage and early osteophyte development in *STR/ort* mice, which develop spontaneous OA, and in collagenase-induced OA rodent models [35]. In contrast, prolonged TGFβ/bone morphogenetic protein 6 (BMP6) expression improves articular cartilage formation [36]. Truncation of the type-II receptor of TGFβ (TGFβRII) promotes terminal chondrocyte differentiation and OA [37]. Specific knock out (KO) of TGFβRII in chondrocytes results in increased expression of *MMP13*, *RUNX2*, and *ADAMTS5* and progressive OA development [32]. In addition, TGFβ expression decreases with age, consistent with the observed increase of OA with age [34]. In contrast, TGFβ seems to aggravate OA in OA joints. OA is attenuated when TGFβ signaling is inhibited in mesenchymal stem cells of subchondral bone or endogenously [30,38,39]. These results indicate that TGFβ protects healthy joints from OA by repressing expression of OA-inducing proteins MMP13, RUNX2, and ADAMTS5, but aggravates the condition of joints with existing OA.

### 2.3. Vitamin D and Osteoarthritis

Whether vitamin D affects healthy joints and OA joints differently, as seems to be the case for TGFβ mentioned above, is not conclusive. Expression of VDR in the articular cartilage of OA patients, but not in that of healthy volunteers, has been reported [40]. This may explain the lack of OA phenotype in long bone joints of VDR KO mice [41] and 1αOHase KO mice [42,43]. The lack of VDR in non-OA cartilage indicates that vitamin D status may influence healthy articular cartilage of long bones indirectly through the endocrine system, rather than directly, despite the presence of vitamin D metabolites in synovial fluid [44]. On the other hand, vitamin D may affect OA cartilage both directly, as evidenced by the presence of VDR in damaged cartilage, and indirectly, through the endocrine system. Therefore, the effect of vitamin D on the initiation and progression of OA must be studied separately.

Furthermore, vitamin D may affect pain and function, radiologic OA, and cartilage volume differently. Therefore, the following review of human (observational and randomized clinical trials (RCTs)) data will focus on the relationship between vitamin D and three different OA outcome measures during the initiation and progression of OA: joint pain and function, radiological OA (determined by JSN and osteophytes), and cartilage volume loss. Relevant animal and cell studies will follow.

#### 2.3.1. Vitamin D and OA in Humans

In humans, no relationship between vitamin D status and OA initiation has been found in those with mean 25(OH)D ≥50 nmol/L for pain, radiologic OA, and cartilage volume loss [45,46,47,48,49,50,51] (Table 1). Elevating vitamin D status may attenuate joint pain in subjects with lower vitamin D status [52]. More research is required to determine the relationship between vitamin D status and initiation of radiologic OA or cartilage volume loss in adults with ‘suboptimal’ 25(OH)D. As for the progression of OA, the literature does not support increasing vitamin intake in participants with 25(OH)D levels ≥50 nmol/L to reduce joint pain, radiologic OA, or cartilage volume loss [46,53,54,55,56,57,58]. However, vitamin D supplementation might benefit those with lower levels of vitamin D by alleviating pain and possibly radiologic OA [57,59,60,61,62]. Altogether, more studies on adults with lower vitamin D status (<50 nmol/L) are needed. The optimal 25(OH)D status for OA is currently unclear, but may be similar to that for bone health, 50 nmol/L [1].

##### Vitamin D and OA Prevention

Joint pain and function: The effect of vitamin D intake or status on the initiation of OA has not been tested by RCTs (Table 1). Only one prospective observational study is available to date. Laslett et al. [52] reported that low vitamin D status (12.5–25 nmol/L) at baseline predicted a five-year change in knee pain and hip pain. No studies on vitamin D status and change in joint function related to OA are currently available. More studies are required to assess the effect of vitamin D status on clinical symptoms of the joint during the initiation of OA.

Radiologic OA: Observational studies in participants with relatively high (≥50 nmol/L) vitamin D status suggest that there is no relationship between vitamin D status and the initiation of radiological OA characterized by osteophytes and JSN [45,47,48,49,50,62]. In Caucasian female twins of the United Kingdom (UK), vitamin D status did not differ between twins with radiological knee OA and those without radiological knee OA (mean 25(OH)D: 86 nmol/L) [45]. On the other hand, in Tasmanian older adults, vitamin D insufficiency (<50 nmol/L) was positively associated with moderate-to-severe JSN [46]. Although the results of cross-sectional studies are conflicting, most prospective observational studies report a null relationship. The incidence of radiographic knee OA was not associated with serum 25(OH)D (mean: 66 nmol/L) or vitamin D intake in the Rotterdam study, a large prospective cohort with a follow-up of 6.5 years [47]. However, an interaction between bone mineral density (BMD) and vitamin D status on OA progression may exist, as subjects with lower lumber spine BMD have increased risk for OA initiation when vitamin D intake and serum levels were low [47]. Among studies with longer follow-up, the Framingham Study cohort [48] and the Framingham Offspring cohort [49] did not detect any relationship between 8- or 9-year knee OA occurrence and serum 25(OH)D at midpoint (~5 years) of the study. The range of 25(OH)D of the lowest tertile in the Framingham cohort was 12.3–60 nmol/L and number of patients was few (*n* = 75) [48]. The Framingham Offspring cohort, of which 87% of the participants did not have OA at baseline, had a mean 25(OH)D status of 49.3 nmol/L during the follow-up period [49]. Incidence of OA in a Finnish population was not associated with a vitamin D status after a 22 year follow-up, however, these participants had high mean vitamin D status (mean 113 nmol/L) [51]. The lack of association between vitamin D status and knee OA patients in prospective studies may be due to the relatively high baseline 25(OH)D or the short follow-up.

Cartilage volume loss: Serum 25(OH)D levels in Tasmanian older men and women without radiographic knee OA or pain were negatively associated with medial and lateral tibial cartilage volume [46]. However, neither baseline (mean: 52.8 nmol/L, range: 13–119 nmol/L) nor change in vitamin D status predicted changes in medial and lateral tibial cartilage volume after 2.9 years of follow-up in subjects without radiographic OA. Thus, vitamin D status may not be associated with initiation of cartilage loss; however, studies in diverse populations and with longer follow up are required.

##### Vitamin D and OA Treatment

Joint pain and function: The association between joint pain/function and vitamin D among OA patients in observational studies differ by study design. Cross-sectional studies indicate a null relationship while prospective studies find a negative relationship between baseline vitamin D status and pain. The lowest tertile of 25(OH)D in the Hertford Cohort Study (range: 17–35.8 nmol/L) had a higher odds ratio of knee pain compared to the highest tertile (range: 51.2–147 nmol/L). However, the association was not statistically significant after adjusting for age, gender, body mass index, season of clinic visit, and severity of radiographic OA [53]. Pain did not differ between Turkish OA patients with 25(OH)D <50 nmol/L and those with 25(OH)D ≥50 nmol/L [54]. In the Tasmanian older adult study, knee pain did not differ between participants whose 25(OH)D levels were consistently ≤50 nmol/L and those whose 25(OH)D levels were consistently >50 nmol/L at baseline, three months, and 24 months [46]. The cross-sectional design of most observational reports is a large limitation, as reverse causation may occur. On the other hand, prospective studies indicate that baseline vitamin D status is associated with change in joint pain. Knee pain was positively associated with baseline and 2.9-year change in 25(OH)D and knee pain at baseline in Tasmanian patients with knee OA [46]. Similarly, baseline vitamin D status <25 nmol/L predicted greater increase in knee pain over five years, although its association with hip pain over 2.4 years was marginal (*p* = 0.08) [52]. Additionally, interaction between vitamin D and other nutrients, in addition to baseline vitamin D status, must be considered. For instance, knee OA patients from two different cohorts with sufficient status/intake of both vitamin K and vitamin D at baseline had better lower-extremity function over 4–5 years of follow-up compared to those who lacked at least one of these two nutrients [63]. Dietary magnesium intake has been shown to be negatively associated with JSN incidence [64] and to regulate vitamin D metabolism [65]. Therefore, not only baseline vitamin D status, but also nutritional status of other nutrients must be considered to understand the association between vitamin D and OA.

Similar to prospective observational studies, RCTs indicate that vitamin D supplementation to patients with 25(OH)D below 50 nmol/L, but not higher, may alleviate pain and improve joint function (Table 2). In the UK, OA patients supplemented with 800 IU/d for three years increased their 25(OH)D level from 52 nmol/L to 76 nmol/L [55]. However, pain, stiffness, and functional loss were not affected. In addition, no interaction was found between baseline vitamin D levels and treatment effect in this study. A post-hoc analysis of the Women’s Health Initiative revealed no effect of calcium (1000 mg/d) plus vitamin D supplementation (400 IU/d) on joint pain or swelling; however, baseline 25(OH)D status was not reported and many were taking supplements on their own [56]. In patients with symptomatic OA, supplementation of 2000 IU/d of vitamin D for two years did not reduce knee pain [57]. Mean baseline 25(OH)D level was >50 nmol/L and >20% of participants had a Kellgren-Lawrence (KL) score of 4, the maximal grade of OA, which may have caused a ceiling effect in this study as OA is currently irreversible. Based on subset analysis, vitamin D supplementation tended to alleviate knee pain in participants with 25(OH)D <50 nmol/L; however, the sample size was too small to achieve statistical significance [57]. The Vitamin D Effect on Osteoarthritis (VIDEO) trial, an RCT which supplemented knee OA patients with 50,000 IU/month for 24 months, resulted in no effect of vitamin D supplementation on pain assessed by the Western Ontario and McMaster Universities Osteoarthritis Index (WOMAC) [58]. On the other hand, an improvement in total WOMAC score and function, but not stiffness, and pain assessed by a visual analog scale was observed with vitamin D supplementation [58]. A major limitation of this study is that though baseline 25(OH)D was relatively low (mean: 43.7 nmol/L), 62% of the participants in the placebo group reached over 50 nmol/L at month 24 [59], which may have masked the effect of the intervention. Through a post-hoc analysis of the study, Zheng et al. [59] found that loss of physical function was less severe in those with consistently higher 25(OH)D levels (>50 nmol/L) compared to those with consistently low (≤50 nmol/L) vitamin D status. The same group found that vitamin D supplementation also suppressed increase in knee effusion-synovitis volume, a measure of joint inflammation which may alter joint function [60]. In line with these results, a pilot RCT performed in India recruited OA patients with 25(OH)D levels ≤50 nmol/L (mean: 37 nmol/L) and provided 60,000 IU/d for 10 days followed by 60,000 IU/month for 12 months [61]. The vitamin D-supplemented group experienced increased serum 25(OH)D levels (mean increase: 45.7 nmol/L) and statistically significant but not clinically meaningful decreases in knee pain and function. These results suggest that the current recommendations for vitamin D intake may alleviate joint pain in OA patients with 25(OH)D levels <50 nmol/L, however, more RCTs in OA patients with baseline 25(OH)D <50 nmol/L are required.

Radiologic OA: Most cross-sectional studies report a positive relationship between vitamin D status and radiologic knee OA, although such a relationship is not reported in prospective observational studies. In one small (*n* = 107) cross-sectional observational study in Turkey, lower vitamin D status (<50 nmol/L) was not associated with higher KL scores [54]. On the other hand, a three-fold higher risk of knee OA progression was associated with lower vitamin D status in the Framingham Study (≤82.5 versus ≥90 nmol/L; *n* = 75), as measured by loss of joint space and osteophyte growth [48]. Larger cross-sectional studies result in higher risk of JSN or osteophyte growth with lower vitamin D status. Participants in the Osteoarthritis Initiative cohort with pre-existing knee OA (*n* = 418) and serum 25(OH)D <37.5 nmol/L had a three-fold higher risk of OA progression as assessed by JSN score [62]. The odds ratio of progressive knee OA was 7.7 in older adults in the lowest tertile of vitamin D intake compared to the highest tertile intake in The Rotterdam Study (*n* = 1248) [47]. Serum 25(OH)D level <50 nmol/L was associated with a greater risk of JSN cross-sectionally, but not, prospectively, in Tasmanian adults (*n* = 880) [46]. The Framingham Osteoarthritis Study (*n* = 715) assessed knee radiographs twice to determine joint space loss, with a mean interval of nine years between each radiograph and a single measurement of 25(OH)D during this interval. Similar to the Tasmanian study, no association between 25(OH)D level (mean: 50 nmol/L) and knee OA worsening was detected [49]. These results indicated that vitamin D status may not influence the progression of radiologic knee OA. 

In line with the results of prospective observational studies, RCTs did not find an effect of vitamin D supplementation on radiologic knee OA progression. The VIDEO study supplemented patients with 800 IU/d for three years which increased 25(OH)D from 62.8 nmol/L to 84.3 nmol/L without affecting JSN [55]. In addition, no interaction was found between baseline vitamin D levels and treatment effects in this study. In a small study performed in US Whites (mean baseline 25(OH)D: 55 nmol/L), supplementation of 2000 IU/d for two years did not affect JSN [57]. These studies indicate that vitamin D supplementation may not benefit OA patients with relatively high vitamin D status (≥50 nmol/L). However, RCTs are limited in patients with 25(OH)D <50 nmol/L or with hip OA. In turn, the null results of vitamin D supplementation in patients with higher vitamin D status indicates that 25(OH)D of 50 nmol/L may be adequate for OA patients.

Cartilage volume loss: Results of prospective observational studies on cartilage volume loss indicate an advantage of vitamin D supplementation. In participants of the Boston Osteoarthritis of the Knee Study, knee OA was assessed at baseline, 15 months, and 30 months by magnetic resonance imaging to detect cartilage loss [49]. Vitamin D status was not associated with OA progression in these participants. Adults with consistently ‘adequate’ levels of 25(OH)D (>50 nmol/L) at baseline, three months, and 24 months experienced less loss of tibial cartilage volume compared to those who maintained 25(OH)D levels ≤50 nmol/L at these three time points [59]. Longitudinal observations over 2.9 years in Tasmanian patients with radiographic knee OA and/or pain revealed a positive association between baseline 25(OH)D level and medial and lateral tibial cartilage volume [46]. 

Results of vitamin D supplementation RCTs in OA patients are relatively consistent in revealing no effect of such supplementation on cartilage volume. McAlindon et al. [57] reported that supplementation of 2000 IU/d vitamin D for two years in patients with symptomatic OA and mean baseline 25(OH)D of 55 nmol/L did not reduce cartilage volume loss. Similarly, Australian OA patients did not benefit from 24 months of vitamin D supplementation (50,000 IU/month) with respect to tibial cartilage volume, tibiofemoral cartilage defects, or change in tibiofemoral bone marrow lesions [58]. However, the effect of vitamin D supplementation may have been reduced by the rise in 25(OH)D level in the placebo group of this cohort (43 % of the placebo group reached >60 nmol/L at three months and 62% of the placebo group reached >50 nmol/L at 24 months) due to factors unrelated to the study protocol [58,59]. The few existing RCTs point to no effect of vitamin D on knee cartilage volume. Studies in those with lower vitamin D status and patients with OA at other sites are needed.

Overall, large prospective studies on the initiation of OA and vitamin D status are lacking, especially in those with relatively lower vitamin D status (<50 nmol/L). Results of cross-sectional studies differ from prospective observational studies, of which follow up is limited to ≤10 years. Several RCTs have been performed in OA patients to evaluate the effect of vitamin D supplementation on OA outcomes. However, patients with relatively high vitamin D status (≥50 nmol/L) and severe OA were included, which may have masked the effect of vitamin D supplementation. Duration of vitamin D supplementation was <3 years for most studies, indicating that long term effects still need to be evaluated.

#### 2.3.2. Vitamin D and OA in Animal Models

Mouse models have been broadly used for OA research due to the ease of care and husbandry, low cost, and relatively short duration to reach skeletal maturity. Although OA may naturally occur in aged C57Bl/6 mice [66], OA research is mostly performed in surgically-induced OA mice. Spontaneous OA also occurs in *STR/ort* mice with age, mimicking naturally occurring (primary) OA which is more prevalent in humans. However, the genetic makeup of this mouse model is not yet fully understood. Thus, caution is needed when interpreting results obtained from these mouse models, especially since the etiology of OA still requires further research. Destabilization of the medial meniscus and anterior cruciate ligament transection are the most commonly used surgical methods to induce OA despite the limitation that they both resemble post-traumatic OA rather than primary OA. Chemical agents such as collagenase, papain, sodium monoiodoacetate, and quinolone are also used to induce OA in animals. Because the pathophysiology of chemically-induced OA is distinct from that of post-traumatic or primary OA seen in humans, these agents are more frequently used to test drug efficacy on OA pain rather than OA etiology. A more detailed review of mouse models in OA can be found elsewhere [67]. Other animal models for in vivo investigation of secondary OA include rabbits [68,69] and equine [70]. The lack of optimal animal models of primary OA adds to the difficulty in understanding the etiology of aging-related OA with respect to vitamin D.

##### Vitamin D and OA Prevention

Currently, there are no reports of OA in long bones of VDR KO mice [41] or 1αOHase KO mice [42,43]. In the mouse temporomandibular joint (TMJ), 1α(OH)ase KO results in a decrease in type-II collagen and an increase in osteoclast number [71]. These results indicate the possibility of different mechanisms between TMJ OA and OA in weight-bearing long bones, such as the knee or hip. In addition, OA was not reported in chondrocyte-specific VDR KO mice using the Col2-cre [72]. Because Col2-Cre is expressed in various chondrocytes in addition to articular chondrocytes, a more articular cartilage-specific Cre should be utilized to clearly understand the local action of vitamin D on joints. In Sprague-Dawley rats, four weeks of vitamin D deficiency resulted in higher Mankin scores by histology, indicating a negative effect of vitamin D deficiency on cartilage, but no effect on subchondral bone [73]. Oral administration of 1,25(OH)_2_D in ovariectomized Sprague Dawley rats on a low vitamin D diet (50 IU/kg diet) protected joints from cartilage erosion in a dose-dependent manner [74]. In addition, expression levels of Tgfβ1 and type-II collagen were increased with higher doses of 1,25(OH)_2_D supplementation. These results point to a weak possibility of preventative effect of vitamin D on animals vulnerable to OA.

##### Vitamin D and OA Treatment

Few studies have reported the role of vitamin D on OA progression in animal models. Daily oral administration of 1,25(OH)_2_D for 20 days post-OA surgery reduced condyle width at a dose of 4 IU/kg body weight (BW)/d, but not at higher doses up to 4000 IU/kg BW/d in male Wistar rats [75]. The intake of 1,25(OH)_2_D did not affect the progression of OA regardless of intake duration. In fact, 1,25(OH)_2_D was effective at the lowest dose (40 IU/kg BW/d) but not at higher doses. The lack of effect with higher doses of 1,25(OH)_2_D may be partially explained by the high vitamin D content in standard chow, which contained 3300 IU/kg diet. The AIN93 diet recommended for rodents contains 1000 IU/kg diet. Even this recommendation is based on weak evidence (i.e., ‘personal communication’) [76]. Some researchers suggest that 1000 IU/kg diet is more than adequate [77]. Well-designed animal studies are required to understand the role of vitamin D in the initiation and progression of OA.

#### 2.3.3. Vitamin D and OA in Cell Models

Thickening of subchondral bone and abnormal bony projections (osteophytes) are prevalent in OA patients. This may indicate the uncoupling of osteoblasts and osteoclasts [78]. Although there seems to be a role of osteoblasts and osteoclasts in OA etiology, as they are responsive to vitamin D, most studies have been performed in chondrocytes.

Cell models for OA are difficult to distinguish between initiation and progression of OA, as chondrocytes hold characteristics of both healthy and OA cartilage. Articular chondrocytes are located between collagen fibers and remain as resting cells following chondrogenesis. The most commonly used chondrocyte cell line, ATDC5, does not have this characteristic of articular chondrocytes, and undergoes differentiation, proliferation, hypertrophy, and apoptosis—characteristics of OA in articular chondrocytes [28]. Murine primary articular chondrocytes can be isolated for mechanistic studies, although the desirable characteristics may last for only one to two passages [79]. In addition, ATDC5 cells and murine primary articular chondrocytes isolated for mechanistic studies express VDR, which is also a characteristic of OA articular chondrocytes. Therefore, specific in vitro results are presented in ‘Vitamin D and OA progression’ section.

Although the use of one type of cell may currently be the only method to study the mechanism of OA in vitro, it may be a very incomplete model due to close involvement of cartilage, synovium, and subchondral bone in OA. The expression of particular proteins or mRNA in two-dimensional (2D) primary culture differs from measurements from cartilage extracts [80]. A large handicap of in vitro studies is the lack of cell models that mimic joint environment. A three-dimensional (3D) culture system may be valuable when applying mechanical load to simulate OA. However, most systems apply sheer stress or tensile stress, which do not precisely imitate the internal environment of the joint [81]. In addition, as the joint is a hypoxic organ, the use of an anaerobic chamber can more closely mimic the joint environment. If vitamin D affects OA initiation and progression through different mechanisms as mentioned above, cell models may reflect only one pathway. As understanding of the etiology of OA increases, better in vitro models may become available.

##### Vitamin D and OA Progression

Research performed in vitro is inconclusive of the effect of vitamin D on OA. In ATDC5 and rat chondrosarcoma chondrocytes, 1,25(OH)_2_D increases the expression of *Mmp13* RNA and protein in a time- and dose-dependent manner, while type-II collagen and aggrecan expression is repressed by 1,25(OH)_2_D [82]. These results indicate that vitamin D may initiate or aggravate OA.

On the other hand, vitamin D may protect OA joints in vivo if it prevents the action of articular cartilage TGFβ, which aggravates the condition of OA joints as seen in other cells. The direct interaction between vitamin D/VDR and TGFβ/SMAD3 has been reported in cell lines [83] and renal [84] and hepatic cells [85], whereas research in chondrocytes is limited. Yanagisawa et al. [83] reported physical interaction of SMAD3 with VDR when bound to 1,25(OH)_2_D and RXR, indicating possible cross-talk between the vitamin D and TGFβ pathways in COS1, HeLa, and HOS cell lines. In the kidney, dietary 1,25(OH)_2_D decreases renal VDR, SMAD3, and bioactive TGFβ in C57Bl/6 mice and Lewis rats [84]. VDR and SMAD3 are coimmunoprecipitated, indicating a physical interaction in the kidney. In hepatic stellate cells, TGFβ induces VDR binding to genes co-regulated by SMAD3, resulting in reduction of SMAD3-occupied liver fibrosis genes [85]. Vitamin D and VDR also regulate MMP13, a downstream target of TGFβ, in bone [86]. However, their roles in articular cartilage and subchondral bone in cartilage destruction remain to be elucidated. In addition, TGFβ may be involved in the regulation of decorin gene expression through VDR, which may play a role in structural organization of the extracellular matrix [87]. These results point to the possibility that vitamin D/VDR ameliorates OA by disturbing the activation of TGFβ pathway or through interaction with SMAD3 or MMP13. However, currently, no studies have been performed to elucidate this mechanism.

#### 2.3.4. Factors that may Influence the Effect of Vitamin D on OA

As with other nutrients and diseases, the effect of vitamin D on OA may differ according to genetics, as observed in its effect on calcium metabolism. Racial differences in vitamin D and calcium metabolism are difficult to explain through the classic vitamin D pathway alone and these differences may consist in OA. For instance, Blacks have lower 25(OH)D levels but higher 1,25(OH)_2_D and bone mass, and thus lower fracture rates, compared to Whites [88]. Asians also have lower 25(OH)D levels [89] but higher calcium retention during adolescence [90] and lower fracture rates [91,92] than Whites. Similarly, the incidence of OA, pain sensitivity, pain inhibition, and function of OA joints differ by race where the occurrence of OA and joint pain is greater in Blacks and Asians than Whites [93,94]. Through a meta-analysis, Zhu et al. [95] reported that the *VDR* Apal polymorphism is associated with OA of the knee, hip, and/or lumbar spine in Asians, but not when Europeans were included. Other well-known *VDR* gene polymorphisms, including Taq1, Fok1, and Bsml, were not associated with OA. Associations between *VDR* polymorphisms and OA have been studied, but few associations between OA and other genes on the vitamin D metabolic pathway have been reported. More studies are required for various races and genes related to the vitamin D pathway.

Although most research on vitamin D and OA has been performed in knee OA, the result may differ in OA at the hip or other regions. In mice, the makeup of hip joint articular cartilage differs from knee joint articular cartilage. One prospective study found that adults with 25(OH)D < 75 nmol/L had a 3.3-fold higher risk of incident JSN, but not osteophytes, within eight years of follow up compared to those with higher 25(OH)D levels [50]. Regarding progression, OA patients with low vitamin D status (≤55 nmol/L) had a greater loss of joint space within eight years compared to those with 25(OH)D ≥ 75 nmol/L [50]. These results, in comparison with the above reviewed knee OA results, suggest the possibility that adequate vitamin D status differs by OA site.

A link between OA and metabolic syndrome (MetS) has been suggested, although the possible mechanisms are unknown [96]. Low vitamin D status, MetS, and OA share obesity as a common risk factor. Individuals with MetS have at least three of the following characteristics—abdominal obesity, hypertension, hyperglycemia, hypertriglyceridemia, and low high-density lipoproteins [97]. A meta-analysis of RCTs resulted in no effect of vitamin D supplementation on glucose homeostasis [98]. However, prospective studies point to a protective role of higher vitamin D status on cardiovascular disease risk [99] and hypertension [100]. Positive associations between MetS or its components and OA have been reported in some observational studies [101,102], though others do not report consistent results [103] and the causal relationship is currently unclear. Relationships among vitamin D, MetS, and OA merits further study and mechanisms remain to be established.

## 3. Conclusions

The effect of vitamin D may differ according to disease state, however, little is understood regarding its effect on the initiation and progression of OA. Whether vitamin D acts on OA joint cartilage locally or through the endocrine system in humans is currently unclear. Clinical observations provide little evidence of a protective effect of vitamin D on cartilage volume loss or radiologic OA initiation, although it might have a preventative effect on joint pain. No RCTs have been performed, while prospective observational studies are limited in their duration to study the role of vitamin D on OA prevention. Regarding joint pain during OA, most trials did not find an benefit of vitamin D supplementation; however, subset analyses and one pilot RCT point to the possibility that patients with low 25(OH)D (<50 nmol/L) may show alleviated joint pain with vitamin D supplementation. Trials on radiologic OA and cartilage loss did not find an effect of vitamin D supplementation in patients with vitamin D status >50 nmol/L, and no trials were performed in patients with relatively low vitamin D status. As most study participants of previous studies were White and the joint of focus was primarily the knee, opportunities for research in other races and joints remain. Research in populations with more frequent OA prevalence such as Asians, Hispanics, and Blacks will expand our knowledge of OA. Various in vitro studies indicate that vitamin D may interact with TGFβ/SMAD3 and inhibit the progression of OA. Unfortunately, adequate cell and animal models are lacking, which hinders the progress of our understanding of OA etiology. The reviewed studies indicate that current evidence is lacking on the effect of vitamin D on the prevention of OA, however, serum 25(OH)D above 50 nmol/L, the target level for bone health, may be adequate for joint health of OA patients as well.

## Figures and Tables

**Table 1:** Observational studies on the relationship between serum 25-hydroxyvitamin D (25(OH)D) and initiation of osteoarthritis (OA) and related symptoms ^1^.

Author {Reference}	*n*	Country (Cohort)	Follow-up (years)	Baseline 25(OH)D (nmol/L)	Results
**Joint pain and function**					
Laslett et al. [51]	764	Australia	5	54	Moderate 25(OH)D (12.5–25 nmol/L) predicted less increase in knee pain
Laslett et al. [51]	765	Australia	2.4	54	Moderate vitamin D status may predict change in hip pain
**Radiographic OA**					
Bergink et al. [47]	1248	Netherlands	8.4	66	No association
McAlindon et al. [48]	556	USA (Framingham Study)	8	74	No association
Felson et al. [49]	277	USA (BOKS)	2.5	51	No association
Felson et al. [49]	715	USA (Framingham Offspring)	9.5	49	No association
Lane et al. [50]	237	USA (SOF)	8	66	No association
Konstari et al. [51]	805	Finland	22	113	No association
**Cartilage volume loss**					
Ding et al. [46]	353	Australia (TASOAC)	2.9	53	No association

^1^ BOKS: Boston Osteoarthritis of the Knee Study; SOF: Study of Osteoporotic Fractures; TASOAC: Tasmanian Older Adult Cohort.

**Table 2:** Randomized clinical trials of vitamin D supplementation on the progression of knee osteoarthritis (OA) and related symptoms ^1^.

				Serum 25(OH)D (nmol/L)			Vitamin D Intervention	Results
			Duration (years)	Baseline	Post-Intervention			
		*n*			Control	Treatment		
**Joint pain and function**								
Sanghi et al. [61]	India	103	1	37	39	83	60,000 IU/d for 10 d followed by 60,000 IU/mo	Vitamin D reduces pain (but unlikely to be clinically relevant)
Arden et al. [55]	UK	468	3	63	61 ^2^	84 ^2^	800 IU/d	No effect of vitamin D
Chelbowski et al. [56]	USA	1911	2	Not reported	Not reported	Not reported	400 IU/d (+1000 mg Ca)	No effect of vitamin D (joint not specified)
McAlindon et al. [57]	USA	146	2	56	62	96	2000 IU/d plus dose escalation to reach over 90 nmol/L	No effect of vitamin D
Jin et al. [58]	Australia	413	2	44	51	84	50,000 IU/mo	No effect of vitamin D
Wang et al. [60]	Australia	340	2	44	51	84	50,000 IU/mo	Vitamin D retards effusion synovitis
**Radiologic OA**								
Arden et al. [55]	UK	427–441 ^3^	3	63	61 ^2^	84 ^2^	800 IU/d	No effect of vitamin D
McAlindon et al. [57]	USA	146	2	56	62	96	2000 IU/d plus dose escalation to reach over 90 nmol/L	No effect of vitamin D
**Cartilage volume loss**								
McAlindon et al. [57]	USA	146	2	56	62	96	2000 IU/d plus dose escalation to reach over 90 nmol/L	No effect of vitamin D
Jin et al. [58]	Australia	413	2	44	51	84	50,000 IU/mo	No effect of vitamin D

^1^ 25(OH)D: 25-hydroxyvitamin D. ^2^ Serum 25(OH)D measures at year 1 of intervention. Mean serum vitamin D status at the end of the intervention (year 3) was not reported. ^3^ Depending on site.
